# The tyrosine phosphatase SHP-2 dephosphorylated by ALV-J via its Env efficiently promotes ALV-J replication

**DOI:** 10.1080/21505594.2021.1939952

**Published:** 2021-06-25

**Authors:** Tuofan Li, Jing Xie, Xiaohui Yao, Jun Zhang, Chunping Li, Dan Ren, Luyuan Li, Quan Xie, Hongxia Shao, Aijian Qin, Jianqiang Ye

**Affiliations:** a Key Laboratory of Jiangsu Preventive Veterinary Medicine, Key Laboratory for Avian Preventive Medicine, Ministry of Education, College of Veterinary Medicine, Yangzhou University, Yangzhou, Jiangsu, China; b Jiangsu Co-innovation Center for Prevention and Control of Important Animal Infectious Diseases and Zoonoses, Yangzhou, Jiangsu, China; c Joint International Research Laboratory of Agriculture and Agri-Product Safety, the Ministry of Education of China, Yangzhou University, Yangzhou, Jiangsu, China; d Institutes of Agricultural Science and Technology Development, Yangzhou University, Yangzhou, Jiangsu, China

**Keywords:** ALV-J, env, SHP-2, phosphorylation, knockout, replication

## Abstract

Avian leukosis virus subgroup J (ALV-J) generally induces hemangioma, myeloid leukosis, and immunosuppression in chickens, causing significant poultry industry economic losses worldwide. The unusual *env* gene of ALV-J, with low homology to other subgroups of ALVs, is associated with its unique pathogenesis. However, the exact molecular basis for the pathogenesis and oncogenesis of ALV-J is still not fully understood. In this study, ALV-J infection and the overexpression of Env could efficiently downregulate the phosphorylation of SHP-2 (pSHP-2) *in vitro* and *in vivo*. The membrane-spanning domain (MSD) in Env Gp37 was the functional domain responsible for pSHP-2 downregulation. Moreover, the overexpression of SHP-2 could effectively promote the replication of ALV-J, whereas knockout or allosteric inhibition of SHP-2 could inhibit ALV-J replication. In addition, the knockout of endogenous chicken SHP-2 could significantly increase the proliferation ability of DF-1 cells. All these data demonstrate that SHP-2 dephosphorylated by ALV-J Env could efficiently promote ALV-J replication, highlighting the important role of SHP-2 in the pathogenesis of ALV-J and providing a new target for developing antiviral drugs against ALV-J.

## Introduction

Avian leukosis virus belongs to the genus of α-retrovirus, the family of retrovirus. According to the characteristics of its *env* gene that encoded viral glycosylated envelope protein (Env), ALV is currently divided into 7 subgroups in chickens: subgroup A, B, C, D, E, J, and K [1–3]. Based on the transmission model, ALV is clustered into the exogenous virus and endogenous virus. Except that subgroup E is a type of endogenous virus with no or low pathogenicity, all the other subgroups are exogenous viruses with different pathogenicity to chickens [4]. Since ALV can cause immunosuppression and different types of tumors in chickens, ALV has caused huge economic losses to the poultry industry throughout the world [5,6]. There is still no effective vaccine or antiviral drug for ALV, and eradication is the only effective way to control ALV. ALV-J was first identified in the UK in 1988, and it mainly causes malignant proliferation of hematopoietic cells, myeloid leukemia, and hemangioma in chickens, which is distinct from other subgroups [1,7]. The *LTR, gag*, and *pol* of ALV-J show more than 90% homology with other ALV subgroups. However, the *env* gene of ALV-J shows only about 40% homology with other ALV subgroups and 97% homology with endogenous EAV HP *env* gene. In contrast, the *env* gene of other ALV subgroups shared 80–85% homology [1,8,9]. ALV-J Env possibly determines its unique pathogenic characteristics. However, little is known about the specific role and molecular basis of Env protein in ALV-J pathogenesis.

ALV-J Env could be further divided into Gp85 on the surface of the cell membrane and Gp37 across the cell membrane [1]. The highly variable Gp85 is mainly responsible for the binding to a viral cell receptor, whereas the conservative Gp37 is responsible for the fusion between virus and cell membrane [10–15]. Our previous study hypothesizes that the Gp37 of ALV-J may be involved in the protein tyrosine signaling pathway via the tyrosine motifs in its cytoplasmic domain (CTD) [15]. Based on the different tyrosine motifs in CTD of Env, ALV-J Env can be clustered into three types (inhibitory, bifunctional, and active Env). Notably, Env protein of ALV-J carrying immune tyrosine-based inhibitory motif (ITIM) might recruit phosphatase of SHP-1, SH2 domain-containing protein tyrosine phosphatase (SHP-2), or SHIP [15]. Thus, ALV-J may further mediate its pathogenesis via these phosphatases. Here, we found ALV-J infection could efficiently induce dephosphorylation of SHP-2 *in vitro* and *in vivo*, and then the MSD within Gp37 of Env was identified to be responsible for decreasing the pSHP-2. Further study revealed that SHP-2 could effectively promote the replication of ALV-J, and the knockout of SHP-2 could significantly increase the proliferation of DF-1 cells.

## Results

### *ALV-J downregulated pSHP-2* in vitro *and* in vivo

**Figure 1.**
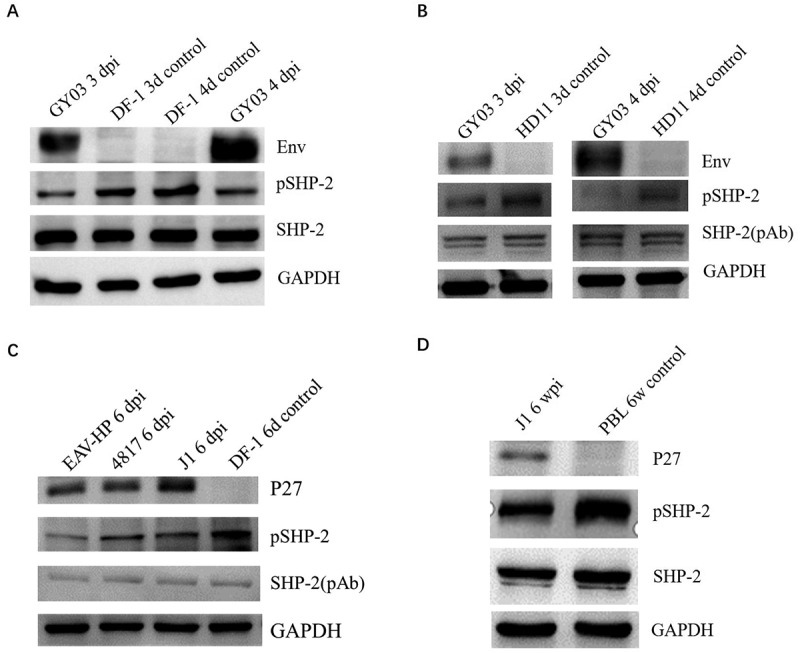
The effect of ALV-J on SHP-2 phosphorylation *in vitro* and *in vivo*. (a) DF-1 cells infected with ALV-J GY03 at an MOI of 1 for 3 or 4d, (b) HD11 cell infected with ALV-J GY03 at an MOI of 1 for 3 or 4 d, (c) DF-1 cells infected with ALV-J different strains at an MOI of 0.1 for 6 d, (d) PBL from SPF chickens infected with ALV-J J1 at 6-week post-infection, and the control PBL were lysed and analyzed by Western blot using the indicated antibodies

Based on our hypothesis, SHP-2 is possibly involved in ALV-J pathogenesis; we first detect the effect of ALV-J on SHP-2 expression. As shown in Figure 1a, ALV-J GY03 strain efficiently downregulated the phosphorylation of SHP-2 but not the expression of SHP-2 in DF-1 cells at 3 d post-infection (dpi) and 4 dpi. A chicken macrophage cell line HD11 was infected with ALV-J further to confirm this in the target cells of ALV-J. Western blot analysis showed a similar result with that in DF-1 cells (Figure 1b). ALV-J J1 and its mutants EAV-HP and 4817 viruses were tested to determine whether different ALV-J strains have the same effect on SHP-2. As shown in Figure 1c, all three viruses caused the downregulation of SHP-2 phosphorylation. The peripheral blood lymphocytes (PBL) from SPF chickens infected with ALV-J for 6 weeks were analyzed by Western blot to investigate the effect of ALV-J infection on SHP-2 *in vivo*. ALV-J could efficiently induce dephosphorylation of SHP-2 in the PBL from chickens infected with ALV-J in comparison with the chickens without the infection of ALV-J as described in Figure 1d. All these demonstrate that ALV-J infection can efficiently dephosphorylate SHP-2 both *in vitro* and *in vivo*.

### MSD in Gp37 of ALV-J Env contributed to the dephosphorylation of SHP-2

**Figure 2.**
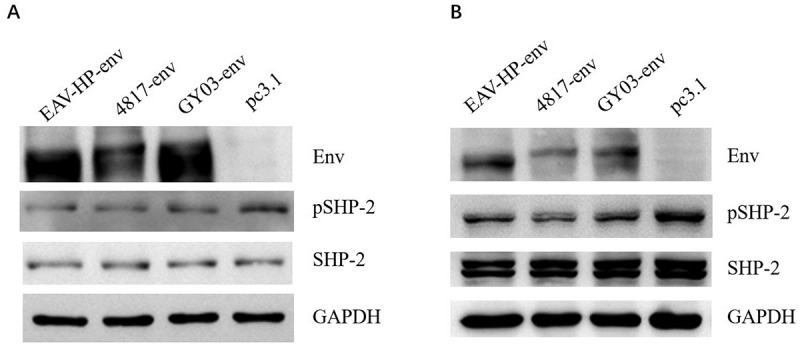
The effect of ALV-J Env on SHP-2 phosphorylation. DF-1 cells (a) and HD11 cells (b) transfected with 4 μg of different types of ALV-J Env plasmids were respectively lysed at 48 hours post-transfection (hpt) and analyzed by Western blot using the indicated antibodies

ALV-J Env is possibly involved in the regulation of SHP-2 expression and phosphorylation, according to our previous hypothesis. Three types of ALV-J Env were transfected into DF-1, and HD11 cells, respectively, and SHP-2 phosphorylation was detected to investigate further whether ALV-J could downregulate pSHP-2 through its Env. The result showed that all three types of Env could induce dephosphorylation of SHP-2 but do not affect the expression level of SHP-2 as described in Figure 2 A and B, which was consistent with that in DF-1 and HD11 cells infected with ALV-J.

**Figure 3.**
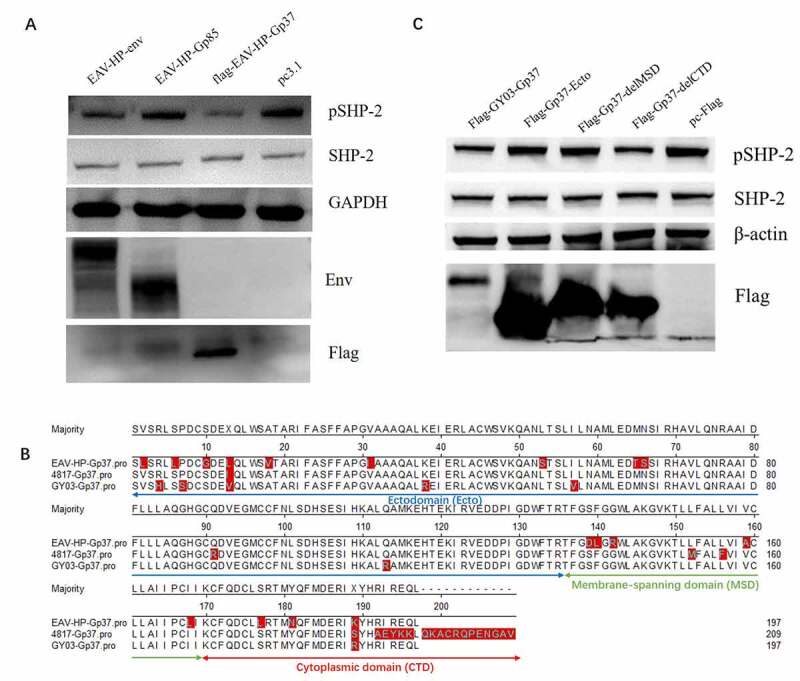
The key domain within ALV-J Env in regulation of SHP-2 phosphorylation. (a) DF-1 cell transfected with 4 μg of EAV-HP-env, Gp85, Gp37 and pc3.1, respectively, (b) DF-1 cell transfected with ALV-J Gp37 and its truncations, respectively, were lysed at 48 hpt and analyzed by Western blot using the indicated antibodies. (c) ALV-J Gp37 sequence structure model diagram. The amino sequence of three types of ALV-J Env were aligned and the sequences of ALV-J Gp37 with blue, green and red line blow them were Ecto, MSD and CTD of ALV-J Gp37, respectively

Since ALV-J Env is composed of Gp85 and Gp37, to find out which domain is responsible for the downregulation of SHP-2 phosphorylation, the effect of Gp85 and Gp37 on the dephosphorylation of SHP-2 was tested in DF-1 cells. As shown in Figure 3a, Gp37, but not Gp85, efficiently downregulated the phosphorylation of SHP-2. Gp37 can be further divided into three domains (i.e. Ectodomain (Ecto), MSD and CTD) (Figure 3b). To further identify the target domain of Gp37 in the regulation of SHP-2, the effect of different truncations of Gp37 on the dephosphorylation of SHP-2 was tested. In the Western blot, we found that the truncations with MSD could downregulate pSHP-2, whereas the truncations lack of MSD could not induce dephosphorylation of SHP-2 (Figure 3c). Thus, ALV-J Env downregulated pSHP-2 via its MSD.

### SHP-2 efficiently promoted viral replication of ALV-J

**Figure 4.**
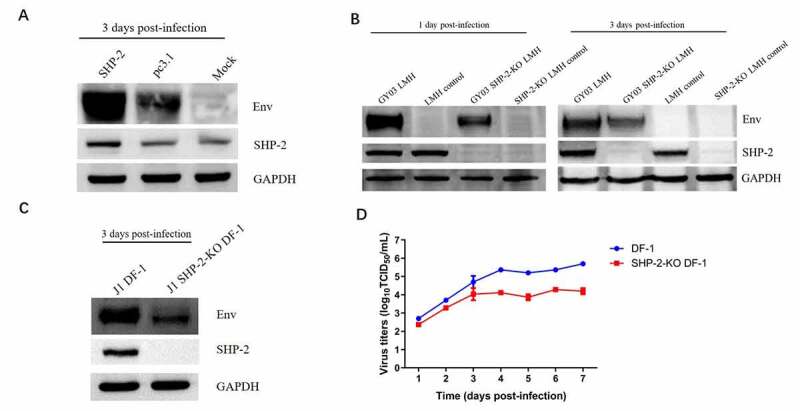
The effect of SHP-2 on ALV-J replication. (a) LMH cells respectively transfected with 4 μg of pc3.1-SHP-2 and pc3.1 were infected with ALV-J GY03 at an MOI of 0.01 at 24 hpt, and then cells were lysed at 3 dpi. (b) LMH cells and SHP-2-KO LMH cells were infected with ALV-J GY03 at an MOI of 0.01 and then cells were lysed at 1dpi or 3 dpi. (c) DF-1 cells and SHP-2-KO DF-1 cells were infected with ALV-J GY03 at an MOI of 0.01 and then cells were lysed at 3 dpi. All the cells were lysed and analyzed by Western blot using the indicated antibodies. (d) Growth curves of ALV-J J1 in DF-1 cells and SHP-2-KO DF-1 cells. ALV-J J1 was inoculated into DF-1 cells and SHP-2-KO DF-1 cells at an MOI of 0.01, respectively, and the supernatants from the infected cells were collected at the indicated time-points for virus titration using TCID_50._

The LMH cells were transfected with chicken SHP-2 and then infected with ALV-J to evaluate the effect of SHP-2 on the viral replication of ALV-J *in vitro*. As shown in Figure 4a, the cells transfected with SHP-2 showed stronger ALV-J viral bands than cells transfected with empty vector. To further confirm this data, LMH and DF-1 cell lines without SHP-2 were first generated through the CRISPR-Cas9 technique, respectively, and designated as SHP-2-KO LMH and SHP-2-KO DF-1. Using the SHP-2-KO LMH and SHP-2-KO DF-1 cell lines, we found that the knockout of SHP-2 in LMH (Figure 4b) and DF-1 cells (Figure 4c) both inhibited ALV-J replication compared to their wild type (WT) cells. Notably, as shown in Figure 4, the viral titers of SHP-2-KO DF-1 cells at 1 to 7 dpi were lower than that of WT DF-1 cells, and the peak titer of SHP-2-KO DF-1 cells was at least 20 times lower than that of WT DF-1 cells (Figure 4d). All these demonstrate that SHP-2 can efficiently promote the viral replication of ALV-J *in vitro*.

### Allosteric inhibition of SHP-2 efficiently decreased the replication of ALV-J

**Figure 5.**
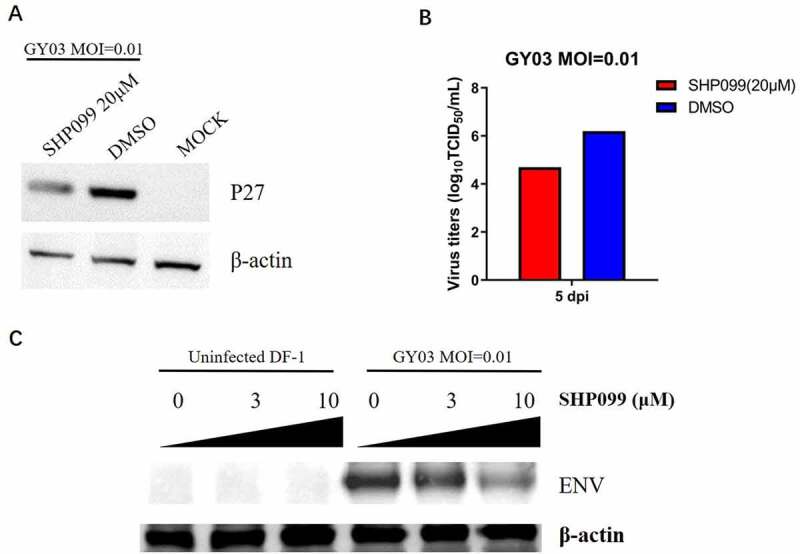
Allosteric inhibition of SHP-2 efficiently inhibited ALV-J replication. DF-1 cells were infected with ALV-J GY03 at an MOI of 0.01, 2 h post infection cell culture medium was replaced with fresh medium with SHP099 or with DMSO. (a) At 3 dpi, the cells were lysed and analyzed by Western blot using the indicated antibodies; (b) At 5 dpi, the supernatants from the infected cells were titrated in DF-1 cells by IFA for TCID_50_; (c) At 3 dpi, the cells cultured with different concentration of SHP099 or DMSO were lysed and analyzed by Western blot using the indicated antibodies

SHP-2 could support the replication of ALV-J; however, the impact of the inhibitor against SHP-2 on ALV-J remains unknown. To verify the possibility of SHP-2 inhibitor as an anti-ALV-J drug, SHP099, an allosteric inhibitor for SHP-2, was selected, which is famous for its high specificity, low toxicity, and high efficiency in cancer therapy [16]. As shown in Figure 5a, the band of ALV P27 from cells treated with SHP099 was weaker than the cell treated with DMSO in the Western blot. Besides, the viral titer of supernatant from cells treated with DMSO was about 150 times higher than that from cells treated with SHP099 (Figure 5b). Notably, the dose dependence test further demonstrated that SHP099 could effectively inhibit ALV-J replication in a dose-dependent manner (Figure 5c). All these data demonstrate that SHP-2 plays a vital role in ALV-J replication and pathogenesis, and the SHP-2 inhibitor SHP099 could be a potential anti-ALV-J drug, indicating that the SHP-2 can be a novel host target for fighting with ALV-J.

### Chicken SHP-2 efficiently regulated the proliferation of DF-1 cells

**Figure 6.**
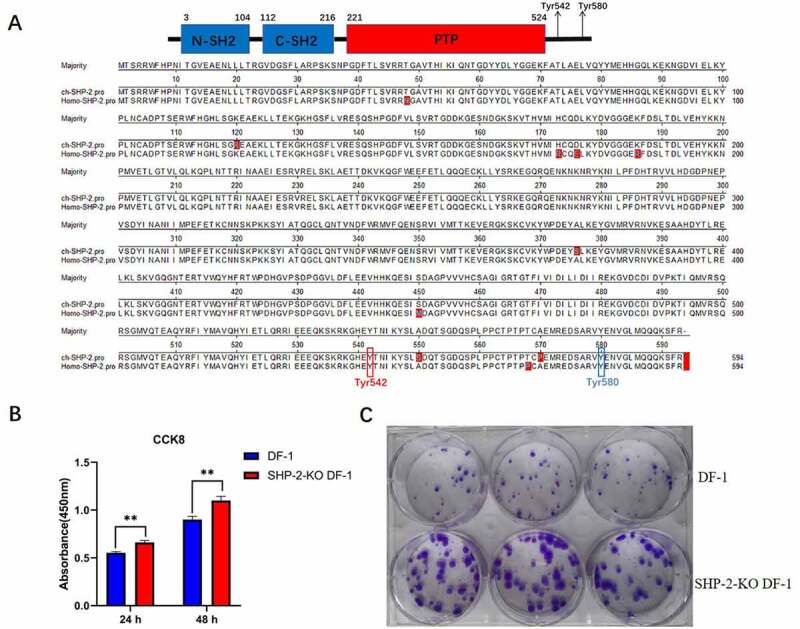
Knock-out of SHP-2 significantly increased the proliferation of DF-1 cells. (a) Sequence structure model diagram and alignment for chicken SHP-2 and human SHP-2. The sequence structure model diagram of chicken SHP-2 showed the important functional domains and tyrosine sites, and the SH2 domains were in blue squares, the PTP domain was in red square, and the tyrosine sites of Y542 and Y580 were indicated with arrows. The amino sequence of chicken SHP-2 and human SHP-2 were compared and aligned. The tyrosine sites of Y542 and Y580 within chicken SHP-2 and human SHP-2 were in the red and blue frames, respectively. (b) 10^4^ cells of DF-1 and SHP-2-KO DF-1 cells were respectively inoculated into 96 well-plate. After cultured for 24 h and 48 h, the cells were respectively analyzed by CCK8 assay as described in the method. (c) Two hundred cells of DF-1 and SHP-2-KO DF-1 cells were respectively inoculated into 6 well-plate and further cultured for 13 d for clonal formation assay. The cells were fixed with 4% paraformaldehyde and stained with 1% crystal violet solution

Sequence analysis of chicken SHP-2 and human SHP-2 revealed that the chicken SHP-2 showed 98.4% homology to human SHP-2 and had the same molecular structure as human SHP-2 (i.e. N-SH2_C-SH2_PTP_Proline rich C-terminal tail) carrying Y542 and Y580 tyrosine phosphorylation sites as described in Figure 6a. Since our study demonstrated that ALV-J infection and the overexpression of ALV-J Env could efficiently downregulate the phosphorylation of SHP-2, SHP-2 could promote the viral replication of ALV-J, it is quite interesting to determine whether chicken SHP-2 can be as a pro-oncogene just like human SHP-2. Thus, we performed the cell counting Kit-8 (CCK8) and clonal formation assay using SHP-2-KO DF-1 cell and WT DF-1 cell. As described in Figure 6b and c, both CCK8 assay and clonal formation assay clearly showed that the knockout of SHP-2 in DF-1 cells significantly increased the cell proliferation compared with WT DF-1 cells. These data demonstrate that the endogenous chicken SHP-2 plays a vital role in controlling cell proliferation, indicating chicken SHP-2 could also be one of the pro-oncogenes in DF-1 cells.

## Discussion

SHP-2 is a nonreceptor tyrosine phosphatase encoded by the *PTPN11* gene. *PTPN11* is also the first proto-oncogene identified as encoding tyrosine phosphatase, one of the hotspots in the cancer research area [17,18]. SHP-2 is generally inactive at resting state, and its N-SH2 domain directly binds to and blocks the active site of the phosphatase domain (PTP), which inhibits its phosphatase activity. However, when its N-SH2 region binds to the tyrosine residue of the substrate protein, its protein conformation will change, thus activating its phosphatase activity [19]. Large numbers of data show that the changes of expression and activity of SHP-2 are closely related to the pathogenesis of a variety of diseases, including hematopoiesis, Noonan syndrome, childhood leukemia, lung cancer, breast cancer, neuroblastoma, and autoimmune diseases [20–25]. Notably, as the key downstream molecule for programmed cell death protein 1 (PD-1), SHP-2 plays an essential role in the negative regulation of T cell activity [26]. In addition, SHP-2 also plays a vital role in regulating viral replication and pathogenesis of several viruses, such as HIV, human respiratory syncytial virus (RSV), poliovirus, human cytomegalovirus (HCMV), and influenza viruses [27–30]. Our previous research hypothesizes that ALV-J Env protein might induce its pathogenesis by recruiting phosphatase of SHP-1, SHP-2, or SHIP via its ITIM located in CTD [15]. In this study, we found that ALV-J could efficiently downregulate the phosphorylation level of SHP-2 at Y542 but not the expression of SHP-2 via its Env protein, whereas the overexpression of SHP-2 could promote the viral replication of ALV-J. As an important tyrosine phosphorylation site in SHP-2, the phosphorylated Y542 triggers the phosphatase activity of the SHP-2 [20,21,23,31]. These findings not only highlight the vital role of SHP-2 in the pathogenesis of ALV-J infection but also provide ALV-J as a good viral model for elucidating the molecular basis of SHP-2 in hematopoiesis, leukemia oncogenesis and immunosuppression.

The regulatory function of SHP-2 on tumor or hematopoietic cells is generally positive. The upregulation of expression or elevation of phosphatase activity of SHP-2 would promote cell proliferation and tumor formation. However, there are also some cases of reverse regulation [23,32–35]. In addition, SHP-2 possesses anti-apoptotic function. It is reported that knockout or knockdown of SHP-2 can increase cell apoptosis and inhibit cell growth [36–39]. The anti-apoptotic function of SHP-2 is extremely important for the homeostasis of human hematopoietic cells, and the imbalance of SHP-2 may lead to disorder of the hematopoietic system [38,39].

It should be noted that the expression of SHP-2 was negatively correlated with the differentiation of hematopoietic stem cells, and the downregulation of pSHP-2 inhibited the proliferation of cells but promoted the differentiation of cells [37]. Notably, in this study, we found that the knockout SHP-2 significantly increased the proliferation of DF-1 cells (Figure 6). Thus, chicken SHP-2 plays a critical role in controlling the proliferation of DF-1 cells, indicating the different roles of SHP-2 in different hosts or cell types. Given that ALV-J mainly induces malignant proliferation of hematopoietic system and immunosuppression, and ALV-J infection downregulates the pSHP-2 *in vivo* and *in vitro*, we hypothesize that the decrease of pSHP-2 induced by ALV-J in hematopoietic cells might break the balance of homeostasis of the hematopoietic cells and subsequently result in immunosuppression, or interfere the differentiation of hematopoietic cells, and finally leading to cell transformation and tumor formation. Besides, Y542 and Y580 are important for SHP-2 tyrosine phosphatase activity and its function. During our study, we not only detected Y542 phosphorylation but also try to detect the phosphorylation of Y580 in DF-1 cells infected with ALV-J. Unfortunately, although the Y580 site is conserved in chicken and homo SHP-2, the commercial monoclonal antibody (mAb) against Y580 of SHP-2 we bought could not detect the target band in DF-1 cells. However, it should be noted that the Y542 and Y580 generally change synergistically, and Y542 is more important for SHP-2 than Y580 [21].

Using different truncations of Env protein, we identified the MSD within Gp37 of ALV-J Env, but not the CTD, was the key domain contributing to the downregulation of the phosphorylation of SHP-2 (Figure 3). Unlike Gp85 protein, the MSD of Gp37 protein is conserved in different ALV-J strains. Only a few mutations but no insertion or deletion were found in MSD of ALV-J. However, this finding is quite unexpected because the ITIM, which potentially recruits phosphatase of SHP-1, SHP-2, or SHIP, is located in the CTD within Gp37 but not in the MSD region. We also checked whether ALV-J and its Env mediated the dephosphorylation of SHP-2 via direct interaction between Env and SHP-2. Unfortunately, we did not find the interaction between them in DF-1 cells by co-immunoprecipitation (data not shown). Therefore, we hypothesized that ALV-J might regulate pSHP-2 by affecting the upstream molecules of SHP-2, like EphA2 or its ligands [31]. Therefore, the molecular mechanism of the MSD of Gp37 on downregulation of the pSHP-2 and the role of the ITIM in the CTD needs to be further elucidated.

No effective vaccine or antiviral drug is currently available for ALV-J, and eradication is the only effective way to control ALV-J. The strict eradication program has been effectively applied in controlling ALV infection in many countries. However, with the continuous spread and variation of ALV-J, more and more different types of domestic chicken flocks are affected, especially in China in the past several years [40–43], challenging the current ALV eradication program. In this study, we revealed that the overexpression of SHP-2 could promote the viral replication of ALV-J, and the allosteric inhibitors SHP099 against SHP-2 could effectively inhibit ALV-J replication with dose-dependence *in vitro*. Since the allosteric inhibitor SHP099 for SHP-2 is famous for its high specificity, low toxicity, and high efficiency in cancer therapy, SHP-2 could be a promising target for fighting against ALV-J. Of course, whether the allosteric inhibitors SHP099 could effectively restrict viral replication and tumorigenesis of ALV-J *in vivo* needs to be further investigated. Besides, since inhibiting SHP-2 by SHP099 could not only inhibit SHP-2 itself but also regulate the downstream pathways, the exact mechanism of SHP099 inhibiting ALV-J remains to be studied.

In conclusion, this is the first demonstration that the chicken tyrosine phosphatase SHP-2 can be dephosphorylated by ALV-J via its Env, whereas SHP-2 promotes ALV-J replication and plays a vital role in controlling the proliferation of DF-1 cells, highlighting SHP-2 can be as a novel target against ALV-J while ALV-J can be as a viral model for elucidating the molecular basis of the diseases associated with SHP-2. Notably, the knockout and overexpression assays demonstrated that WT SHP-2 (especially endogenous SHP-2) is beneficial for ALV-J replication. Thus, the impact of SHP-2 on ALV-J replication was possibly not only associated with its Y542 tyrosine site but also its expression and protein conformation (active or inactive). So, to our knowledge, decreasing the expression of SHP-2 would be more efficient than only inhibiting the phosphorylation of SHP-2, especially for research on anti-ALV-J in the future. Of course, the roles of SHP-2 truncations and tyrosine site mutants on ALV-J replication need to be elucidated in the future. In addition, it is unclear whether the downregulation of pSHP-2 is caused by signal disorder mediated by ALV-J, which destroys the balance of the hematopoietic system and leads to tumor or immunosuppression, or whether the host cells perceive ALV-J infection and play a counter-regulatory role in the process of resisting ALV-J infection. However, as an important tyrosine phosphatase of the host, the effect of ALV-J and Env on pSHP-2 and the impact of SHP-2 on ALV-J both undoubtedly indicate that SHP-2 plays an important role in ALV-J pathogenesis. Further studies should focus on how ALV-J and its Env interact with SHP-2 or SHP-2-related signaling pathways and further mediates its pathogenesis and oncogenesis, which is of great significance to reveal the pathogenesis of ALV and other retroviruses or oncogenic viruses.

## Materials and methods

### Cells and plasmids

DF-1 cells (from ATCC, kept in our lab) were cultured in Dulbecco’s modified Eagle’s medium (DMEM) with 5% fetal bovine serum (FBS). LMH cells (from ATCC, kept in our lab) were cultured in DMEM/F12 with 10% FBS. HD11 cells (from The Pirbright Institute, UK) were cultured in 1640 medium with 10% FBS. All the cell mediums were supplied with 100 U/ml penicillin and 0.1 mg/ml streptomycin. DF-1 and LMH cells were cultured in a cell incubator with 5% CO_2_ at 37°C, whereas HD11 cells were culture at 41°C. pcDNA3.1-SHP-2 was previously constructed and preserved by our group [44]. pcDNA3.1-EAV-HP-env, pcDNA3.1-GY03-env, and pcDNA3.1–4817-env, which contained the *env* gene from endogenous avian retrovirus EAV-HP (GenBank accession number AF125528), ALV-J GY03 strain (GenBank accession number GU982308), and the ALV-J 4817 strain (GenBank accession number AF247385), respectively, were maintained in our lab. pcDNA3.1-EAV-HP-env, pcDNA3.1-GY03-env and pcDNA3.1–4817-env express inhibitory, bifunctional and active Env, respectively [45]. All the ALV-J Env truncations in the pcDNA3.1 backbone were constructed using the plasmids we mentioned above via homologous recombination technique (Vazyme, Nanjing, China) using the primers listed in Table 1. The ALV-J infectious clone J1 with a bifunctional *env* gene was a generous gift from Yixin Wang, Shangdong Agriculture University.

**Table 1.** Primers for construction of ALV-J Env truncations

Target sequence	Template/Primers for amplifying target gene fragments	Template/Primers for amplifying linearized vectors
EAV-HP-Gp85	pcDNA3.1-EAV-HP-env/EAV-HP-gp85F: agcttggtaccgagc ATGGACCAAGTCATTAAGG; EAV-HP-env-gp85R: atatctgcagaattc TTAGCGCCTGCTACGGCGGTGAC	pcDNA3.1/pcDNA3.1_F: gaattctgcagatatCCAGCACAGTG pcDNA3.1_R: gctcggtaccaagctTAAGTTTAAACG
Flag- EAV-HP-Gp37	pcDNA3.1-EAV-HP-env/EAV-HP-env-gp37F: agcttggtaccgagcATGGATTACAAGGATGACGACGATAAGTCGCTGAGTCGTCTCTTGCCTG EAV-HP-env-gp37R: atatctgcagaattc CTACAGCTGCTCCCTAAT	pcDNA3.1/pcDNA3.1_F: gaattctgcagatatCCAGCACAGTG pcDNA3.1_R: gctcggtaccaagctTAAGTTTAAACG
Flag-GY03-Gp37	pcDNA3.1-GY03-env/GY03-env-gp37F: agcttggtaccgagcATGGATTACAAGGATGACGACGATAAGTCGGTGAGTCATCTCTCGTCTG GY03-env-gp37R: atatctgcagaattc ctacagttgctccctaattctatg	pcDNA3.1/pcDNA3.1_F: gaattctgcagatatCCAGCACAGTG pcDNA3.1_R: gctcggtaccaagctTAAGTTTAAACG
Flag-GY03-Gp37-ecto	pcDNA3.1-Flag-GY03-gp37/Flag-G-gp37-F: agcttggtaccgagcATGGATTACAAG G-gp37-ecto-R: atatctgcagaattcCTACGTGCGCGTAAACCAATCCCCTATG	pcDNA3.1/pcDNA3.1_F: gaattctgcagatatCCAGCACAGTG pcDNA3.1_R: gctcggtaccaagctTAAGTTTAAACG
Flag-GY03-Gp37-delMSD	pcDNA3.1-Flag-GY03-gp37/Flag-G-gp37-F: agcttggtaccgagcATGGATTACAAG G-gp37-delMSD-R: atcctggaagcacttCGTGCGCGTAAACCAATCCCCT	pcDNA3.1-Flag-GY03-gp37/Line-G-gp37-delMSD-F: aagtgcttccaggattgcctatcgag pcDNA3.1_R: gctcggtaccaagctTAAGTTTAAACG
Flag-GY03-Gp37-delCTD	pcDNA3.1-Flag-GY03-gp37/Flag-G-gp37-F: agcttggtaccgagcATGGATTACAAG G-gp37-delCTD-R: atatctgcagaattcCTAGATTATACATGGAATGATAGCTAATAG	pcDNA3.1/pcDNA3.1_F: gaattctgcagatatCCAGCACAGTG pcDNA3.1_R: gctcggtaccaagctTAAGTTTAAACG

### Antibodies

Mouse mAb JE9 against Gp85 of ALV-J Env [46] and mouse mAb 5D3 against ALV P27 [47] were preserved in our lab. An anti-chicken SHP-2 polyclonal antibody was previously generated by our group [44]. Anti-SHP-2 mouse mAb was purchased from BD Biosciences. Anti-SHP-2 Y542 rabbit mAb was purchased from Abcam. Anti-GAPDH and anti-β-actin mAbs were purchased from Abclonal. Anti-Flag rabbit polyclonal antibody was purchased from Proteintech Group.

### Cell transfection

DF-1 and LMH cells were inoculated into a 6-well plate at about 80% confluence, and cells were transfected using Mirus-TransIT transfection reagent. For the HD11 cell line, cells were transfected using Lipofectamine 3000 reagent (Thermo). At 6 h post-transfection, cell supernatants containing transfection reagents were replaced with a fresh culture medium.

### Construction of plasmid for Genome edit

The sgRNA for knockout of chicken SHP-2 was designed with an online tool (www.benchling.com) and then cloned into a CRISPR/Cas9 Plasmid LentiCRISPR v2. The sgRNA sequences targeting SHP-2 are F: 5ʹ- CACCGGTTTCATCCAAATATCACTG −3ʹ, R: 5ʹ- AAACCAGTGATATTTGGATGAAACC-3ʹ.

### Generation of SHP-2-KO cell lines by CRISPR/Cas9

LMH and DF-1 cells were respectively cultured in a 6-well-plate at about 80% confluence and then transfected with gRNA targeting chicken SHP-2. The cells were screened by puromycin for 10 d and subsequently serial-diluted to obtain a monoclonal cell line. The generated SHP-2-KO cell lines were further confirmed by sequencing and Western blot.

### Virus infection

ALV-J JS09GY3 was isolated and preserved in our lab [48]. ALV-J J1, EAV-HP, and 4817 viruses were previously rescued and maintained in our lab [45]. DF-1, LMH, and HD11 cells were seeded into a 6-well-plate at about 70% confluence and cultured for 16 h. The cells were infected with the indicated viruses at MOI of 1, 0.1, or 0.01. At 2 h post-infection (hpi), the cells were washed with PBS, and fresh DMEM with 1% FBS was added to maintain the cells.

### Isolation of chicken PBL

The 1-d-old SPF chicken infected with ALV-J J1 or inoculated with PBS were introduced elsewhere [45]. The PBL from chickens infected with J1 at 6 weeks post-infection and the PBS control were isolated using a chicken peripheral blood lymphocyte isolation kit (Solarbio, Beijing, China). About 2 × 10^6^ cells from each group were lysed and further tested by Western blot.

### Viral growth curves in DF-1 and SHP-2-KO cell lines

The viral replication kinetics were measured as previously described [49]. In brief, DF-1 and SHP-2-KO DF-1 cells in 6-well plates were respectively infected with ALV-J at an MOI of 0.01. Two hundred microliters from the infected cell supernatants were collected at the indicated time points, and 200 μL fresh medium was added to each well. The viral titers were titrated in DF-1 cells by IFA, and their TCID_50_ was calculated using the Reed–Muench method. GraphPad Prism 5 software was used to construct the final viral growth curves.

### Virus inhibition assay using SHP-2 allosteric inhibitor

DF-1 cells in 6-well plates were infected with ALV-J at an MOI of 0.01. Two hours post-infection, the infected cells were washed with PBS. Then, the cells were cultured with 1% FBS DMEM with SHP-2 allosteric inhibitor SHP099 (MedChemExpress) in the indicated concentration or with DMSO solvent in the same volume. Three days post-infection, the viruses in the supernatants of the infected cells were titrated, and the cells were lysed and analyzed by Western blot.

### Indirect fluorescence assay (IFA)

DF-1 cells infected with ALV-J were fixed with prechilled acetone-ethanol (3:2) solution for 10 min and washed once with PBS. Then, the plates were incubated using mAb JE9 for 45 min. After washed with PBS, the plates were further incubated using goat anti-mouse labeled-fluorescein isothiocyanate (FITC) for 45 min again. After washed with PBS, the cells were observed using an inverted fluorescence microscope.

### Western blot

Cells were lysed on ice for 30 min using cell lysis buffer (CST). Then, the lysates were centrifuged at 12,000 rpm at 4 C, and the supernatants were collected and boiled for 10 min with protein loading buffer. After separation via SDS-PAGE, the denatured samples were transferred onto nitrocellulose membrane (NCs) for Western blot. After blocking with 5% nonfat milk or BSA in PBST for 1.5 h at room temperature (RT), the NCs were incubated with the indicated antibodies 2 h at RT or overnight at 4°C. After three washes with PBST, the NCs were incubated with HRP-conjugated anti-mouse or rabbit IgG (diluted in 1:15000). After three washes, the NCs were incubated with Enhanced Chemiluminescent and developed using Tanon 5200 chemiluminescence image analysis system.

### CCK8 assay

DF-1 and SHP-2-KO DF-1 cells were digested by 0.25% trypsin/0.02% EDTA and then suspended with 10% FBS DMEM medium. Then, 10^4^ cells of each cell line in 100 μL culture medium were seeded into 96-well-plate with three duplicates and cultured for 24 and 48 h. Ten microliters of CCK-8 solution (MedChemExpress) were added into each well and incubated for 4 h at 37°C. The plate was gently mixed using a shaker, and the OD_450_ value was read using a microplate reader.

### Clonal formation assay

DF-1 and SHP-2-KO DF-1 cells were digested by 0.25% trypsin/0.02% EDTA and then suspended with 10% FBS DMEM medium. Then, 200 cells of each cell line were seeded into a 6-well-plate with three duplicates. Fresh medium was used to replace every 5 ds until day 13. Cells were fixed with 4% paraformaldehyde and stained with 1% crystal violet solution before observation.

### Statistical analysis

The statistical results are presented as means ± standard deviations. The statistical analysis in this study was performed with a Student *t*-test using GraphPad 5 software. A P value of 0.05 was considered significant. * and ** indicate *P* values of less than 0.05 and 0.01, respectively.

## Data Availability

The datasets used and/or analyzed during the current study are available from the corresponding author on reasonable request.
